# Metabolic reprogramming in radiation-induced heart disease: the emerging role of PET imaging

**DOI:** 10.3389/fcvm.2026.1883964

**Published:** 2026-07-23

**Authors:** Yifan Zhao, Jia Li, Shihao Huangfu, Feiyu Wang, Sanlin Wang, Bingpan Wang, Yanxia Zhang, Caixia Jin, Tianyi Wang, Zijian Wang, Jun Wu

**Affiliations:** 1 College of Basic Medicine, Shanxi Medical University, Taiyuan City, Shanxi, China; 2 Department of Nuclear Medicine, Shanxi Fenyang Hospital, Lüliang City, Shanxi, China; 3 Graduate School, Shanxi Medical University, Taiyuan City, Shanxi, China

**Keywords:** metabolic reprogramming, molecular imaging, myocardial metabolism, positron emission tomography, radiation-induced heart disease

## Abstract

Radiation therapy is widely used to treat various cancers, but it can lead to radiation-induced heart disease, which is the main cause of long-term morbidity and mortality in cancer survivors. Traditional imaging methods, such as echocardiography and cardiac magnetic resonance, often find structural or functional changes in the late stage of the injury, missing early subclinical injury. This narrative review summarizes the evidence from positron emission tomography studies and shows that molecular imaging can identify early metabolic reprogramming in the radiation-induced heart disease, including the shift from fatty acid oxidation to increased glucose utilization, as well as microvascular dysfunction and fibroblast activation. Tracers like fluorodeoxyglucose and fibroblast activation protein inhibitors may be able to detect metabolic inflammation and active fibroblast remodeling before obvious structural changes. In radiation-induced heart disease, myocardial fluorodeoxyglucose uptake needs to be interpreted according to different stages. There are, however, still challenges remain, including physiological myocardial fluorodeoxyglucose uptake, lack of standardized imaging protocols, limited prospective data and the need to reduce cumulative radiation exposure. Future research should focus on large multicenter longitudinal studies, hybrid positron emission tomography and magnetic resonance imaging, risk stratification based on artificial intelligence to integrate molecular imaging into conventional cardiac tumor monitoring and achieve timely cardiac protection interventions.

## 1 Introduction

Radiation therapy (RT) serves as a cornerstone in the multimodal management of various intrathoracic malignancies, ranging from esophageal carcinomas and mediastinal lymphomas to breast malignancies and non-small cell lung cancer (NSCLC) (1). Driven by the clinical integration of high-precision radiation modalities, especially proton beam therapy and intensity-modulated radiation therapy, patient survival has improved. However, this progress has also led to increasing concern about treatment-related toxicity (2). Among these, Radiation-Induced Heart Disease (RIHD) has evolved from a rare clinical complication into one of the main causes of non-cancer-related morbidity and mortality in cancer patients (3). The clinical manifestations of RIHD are diverse, including a series of complex cardiovascular dysfunctions (4). These pathological changes include not only the acceleration of coronary artery atherosclerosis and myocardial fibrosis but also some structural damage, such as heart valve dysfunction and pericardial effusion, which may eventually develop into congestive heart failure (5–7). One of the major challenges of RIHD is its prolonged subclinical course. Heart injury will gradually accumulate over time and there may be no obvious symptoms for many years or even decades in clinical practice (6, 8). This delay causes RIHD to be a long-term health problem. It significantly reduces the quality of life of patients and the overall survival rate. Therefore, early detection and timely intervention are critical to mitigate these late-stage consequences (9).

Currently, the monitoring strategy of cardiac toxicity mainly relies on traditional imaging methods, particularly echocardiography and cardiac magnetic resonance imaging (CMR) (10). These tools are indispensable in clinical practice. However, they are mainly used to detect structural abnormalities or overall function decline. Widely used parameters, for example, left ventricular ejection fraction (LVEF), usually reflect relatively advanced myocardial damage. Therefore, these indicators lack the sensitivity required for early detection (11, 12). It should also be acknowledged that modern echocardiography techniques, especially speckle-tracking based global longitudinal strain (GLS), can detect subclinical myocardial dysfunction before a noticeable drop in LVEF (13). Advanced CMR methods, including late gadolinium enhancement, T1/T2 mapping, and extracellular volume quantification, also improve the detection of myocardial edema, diffuse fibrosis, and interstitial remodeling (14). Even more sensitive techniques, such as late gadolinium enhancement and parametric mapping, often capture downstream tissue changes rather than the earliest biological alterations (15). Therefore, there remains a gap in identifying the early and reversible stages of subclinical cardiac injury. This gap limits the opportunity for patients receiving chest radiotherapy to get timely intervention.

More and more evidence indicates that lesions in RIHD begin at the molecular and cellular levels before structural remodeling (9, 16, 17). In particular, radiation-induced endothelial damage and mitochondrial dysfunction will disrupt the myocardial energy balance, shifting in substrate utilization from fatty acid oxidation to increased glucose metabolism (16, 18, 19). This process is called metabolic reprogramming. It is a response to impaired energy supply and chronic low-grade ischemia. Importantly, these metabolic changes may occur before detectable changes in perfusion, structure, or function, highlighting their potential role as early indicators.

Positron emission tomography (PET) provides a unique noninvasive imaging modality to detect these early pathological changes (20). Its specific advantage is that it can assess molecules and functions that LVEF, GLS, or CMR tissue characterization can't directly measure. By evaluating indicators such as myocardial metabolism, perfusion, and fibroblast activation, PET can provide insights beyond traditional imaging. Tracers such as Fluorine-18 fluorodeoxyglucose (^18^F-FDG) can reflect glucose utilization and inflammatory activity, while perfusion tracers are used to evaluate microvascular function. In addition, emerging tracers such as fibroblast activation protein inhibitor (FAPI) can detect activated fibroblasts associated with early fibrosis remodeling.

Although previous reviews have discussed RIHD mechanisms and nuclear medicine imaging, the relationship between metabolic reprogramming, disease stage, and the interpretation of different PET tracers remains insufficiently addressed. This narrative review summarizes the current evidence on PET-detectable metabolic and cellular changes in RIHD and discusses the roles of FDG, perfusion PET, and FAPI PET in identifying early myocardial injury, microvascular dysfunction, and fibroblast activation. It also considers practical factors that may influence PET findings, including imaging timing, myocardial preparation, radiation dose, patient characteristics, and quantitative methods, which may partly account for differences among previous studies.

### 1.1 Comparison with previous reviews and scope of the present review

Previous reviews of RIHD have mainly focused on its general pathophysiology, clinical manifestations, screening, and management (3, 4, 6). Nuclear medicine methods have also been reviewed, particularly myocardial perfusion and metabolic imaging after thoracic radiotherapy (20). These studies provide an important basis for understanding RIHD. However, the relationship between metabolic changes, disease progression, and the interpretation of different PET tracers has not been discussed in detail. To clarify the differences in scope and emphasis, previous reviews are compared with the present review in Table 1.

**Table 1:** Comparison of representative previous reviews with the present review.

Previous reviews	Main focus	Difference from the present review
Sárközy et al. (6) and Wang et al. (4)	Pathophysiological, cellular, and molecular mechanisms of RIHD, with discussion of possible preventive and therapeutic approaches	The present review links these mechanisms with PET findings related to glucose metabolism, myocardial perfusion, and fibroblast activation.
Mehta et al. (3)	Clinical manifestations, screening, diagnosis, and management of RIHD	The present review focuses more specifically on early molecular and functional changes that may be detected by PET before obvious structural or functional abnormalities develop.
Polomski et al. (20)	Nuclear medicine imaging of radiation-induced cardiotoxicity, including myocardial perfusion and metabolic imaging	The present review places metabolic reprogramming at the center of the discussion and further emphasizes stage-dependent FDG findings, recent FAPI evidence, and the complementary roles of different PET tracers.
Present review	PET imaging of metabolic and cellular changes in RIHD	Integrates FDG, perfusion PET, and FAPI PET with the underlying pathological processes, and discusses their potential roles in early detection, risk assessment, and longitudinal monitoring.

The present review has a narrower focus. We use metabolic reprogramming as the main link between radiation-induced myocardial injury and PET findings. In particular, we discuss glucose metabolism, microvascular dysfunction, and fibroblast activation together, rather than treating FDG, perfusion PET, and FAPI PET as separate imaging techniques. We also emphasize that myocardial FDG uptake may vary with the stage and severity of injury. Increased or heterogeneous uptake may be seen during active inflammation and enhanced glucose utilization, whereas reduced uptake may occur in areas with severe tissue injury, loss of viable cardiomyocytes, or fibrotic replacement.

In addition, this narrative review includes recent evidence on FAPI PET and discusses possible reasons for the different findings reported in previous FDG studies, including imaging timing, myocardial preparation, radiation dose, and study population. We also summarize practical issues in patient preparation, image analysis, and reporting. The aim is to help readers interpret PET findings in relation to the underlying biological process and to identify areas that require further prospective study.

## 2 Literature search and selection

In April 2026, we conducted a comprehensive literature retrieval in four major databases: PubMed, Web of Science, Cochrane Library, and Europe PMC. The search covered publications from January 2021 to April 2026, using keywords related to radiation-induced heart disease, PET molecular imaging, and radiotherapy. Limiting the search to publications from January 2021 onward is to focus on the cutting-edge developments in molecular imaging technology and reflect the latest clinical evidence in modern radiotherapy practice. Priority was given to high-quality original clinical studies. This study defines that as: prospective or retrospective cohort studies, clinical trials, or well-designed translational animal model studies, which need to have a complete trial protocol and be directly related to PET imaging of RIHD. Case reports, conference abstracts, reviews and studies that do not use core cardiovascular imaging indicators as study endpoints are excluded. Final inclusion of studies was determined by consensus among the authors. The detailed search strategies for all databases are provided in Supplementary Material 1.

## 3 Pathophysiology of RIHD

**Figure 1:**
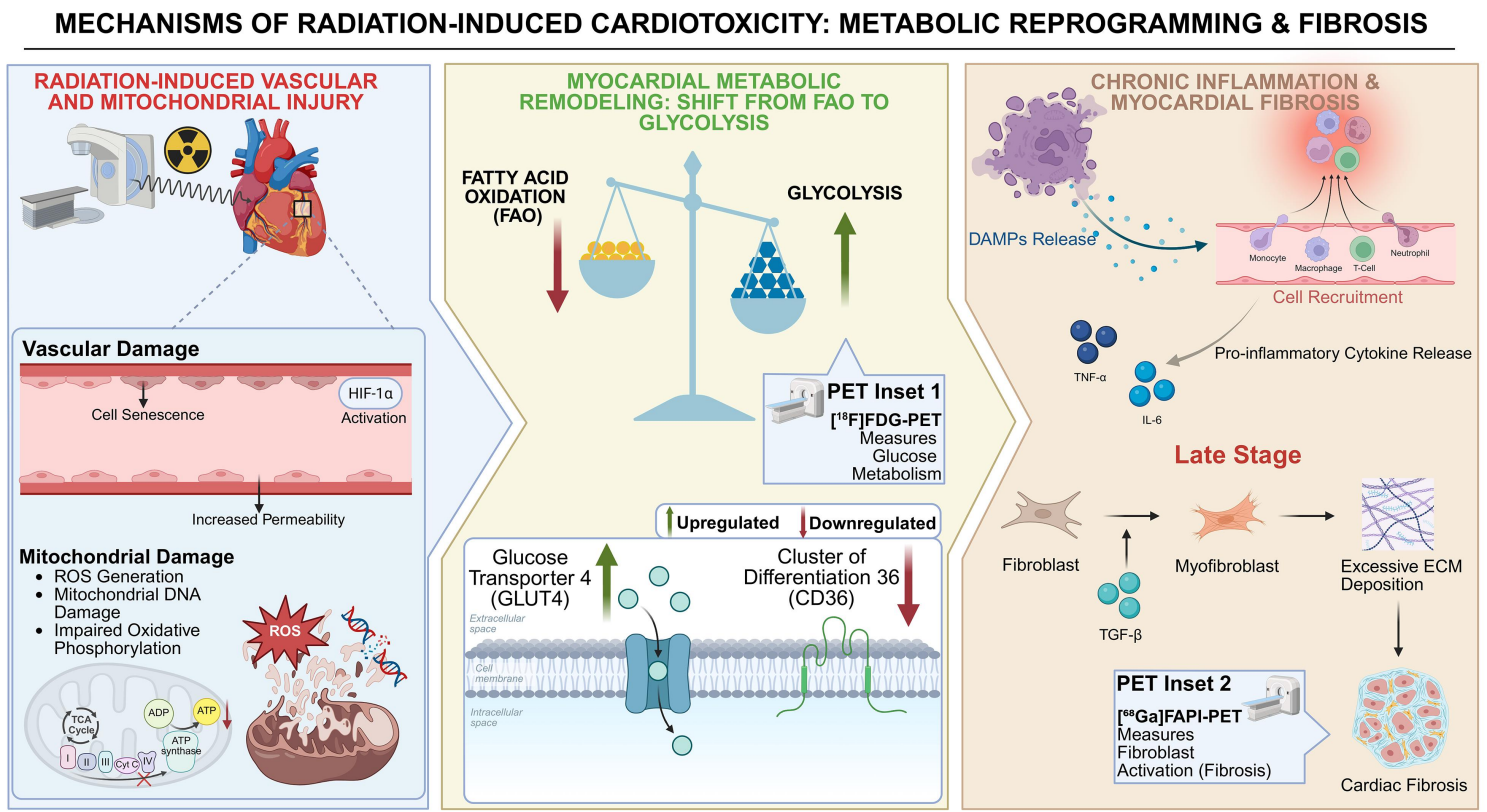
Schematic illustration of the mechanisms in radiation-induced heart disease (RIHD). Ionizing radiation can cause damage to the endothelium and microvascular dysfunction, leading to cell apoptosis and increased capillary permeability. At the same time, mitochondrial damage triggers oxidative stress and decreases ATP production. These early injuries drive metabolic reprogramming, shifting the body from fatty acid oxidation to glucose utilization. Inflammatory activation further worsens metabolic disorders and tissue damage. Over time, persistent damage promotes fibroblast activation, excessive extracellular matrix deposition, and myocardial fibrosis, resulting in cardiac structural remodeling and functional decline. RIHD, radiation-induced heart disease; HIF-1*α*, hypoxia-inducible factor 1-alpha;ROS, reactive oxygen species; ATP, adenosine triphosphate, DNA, deoxyribonucleic acid; PET, positron emission tomography; FAO, fatty acid oxidation; ECM, extracellular matrix; GLUT4, glucose transporter 4; CD36, cluster of differentiation 36;[^18^F] FDG, Fluorine-18 fluorodeoxyglucose; DAMPs, damage-associated molecular patterns; TNF-α, tumor necrosis factor-alpha; IL-6, interleukin-6; TGF-β, transforming growth factor-*β*; [⁶⁸Ga] FAPI, gallium-68 fibroblast activation protein inhibitor. Created with BioRender (https://biorender.com).

Current evidence shows that RIHD is caused by interrelated events involving microvascular damage, mitochondrial dysfunction, metabolic remodeling and chronic inflammation, rather than being driven by a single pathway (4, 9, 17). The pathological progression of RIHD is shown in Figure 1, which systematically shows the responses from early molecular and cellular damage after ionizing radiation exposure to late heart structural and functional abnormalities, highlighting the complex interactions among key factors like microvascular damage, mitochondrial dysfunction, metabolic remodeling, chronic inflammation, and progressive fibrosis.

### 3.1 Endothelial injury and microvascular dysfunction

When exposed to ionizing radiation, vascular endothelium becomes one of the main and most direct cell targets. Radiation exposure can trigger Deoxyribonucleic Acid damage, increases oxidative stress, and promote premature aging, ultimately impairing the integrity and function of the endothelium (21, 22). As a result, nitric oxide bioavailability decreases, while pro-inflammatory signaling pathways are activated (22, 23). With the chronic progression of vascular injury, the aforementioned pathological changes trigger progressive microvascular rarefaction and diminished capillary density, all of which culminate in severely compromised myocardial perfusion (24). Even without significant epicardial coronary artery disease, this microvascular dysfunction can lead to chronic ischemia. Under the conditions, hypoxia-related pathways, particularly Hypoxia-inducible factor 1-alpha (HIF-1*α*) signaling, become activated and promote a shift toward glycolytic metabolism (25).Importantly, this imbalance between perfusion and metabolism provides a biological basis for imaging abnormalities (26). An experimental study had shown that metabolic changes can precede structural damage and may be detectable by PET imaging techniques assessing myocardial perfusion and glucose utilization (18).

### 3.2 Mitochondrial damage and oxidative stress

Mitochondria play a pivotal role in maintaining a steady state of cardiac energy and are particularly sensitive to radiation exposure (27). This damage will destroy the electron transfer chain, which will lead to damage to oxidative phosphorylation and reduce the production of Adenosine Triphosphate (ATP) (4, 28). At the same time, dysfunctional mitochondria will produce excessive reactive oxygen species (ROS). These will further aggravate cell damage and thus form a vicious circle of oxidative stress (16, 29). This process impairs the viability and vitality of myocardial cells while disrupting normal substrate utilization, particularly fatty acid oxidation.

The resulting energy imbalance is closely related to the changes in myocardial metabolism. From the perspective of molecular imaging, this kind of mitochondrial dysfunction is of great value. Because it leads to regional heterogeneity in metabolic activity, which may be displayed as abnormal tracer uptake patterns on PET.

### 3.3 Metabolic remodeling

One of the main characteristics of RIHD is the gradual loss of metabolic flexibility (30). Under normal physiological conditions, the adult heart mainly relies on fatty acid oxidation to produce energy (31, 32). However, radiation exposure will inhibit key lipid metabolism pathways while enhancing glucose utilization. This change is mediated by multiple mechanisms, including the downregulation of the peroxisome proliferator-activated receptor-α (PPAR-α) signaling and activation of the hypoxic reaction pathways (33). Specifically, inhibiting PPAR-α will reduce the expression of some key downstream targets, like carnitine palmitoyltransferase-1 (CPT-1) and fatty acid transport protein (CD36), which seriously weakens the heart's uptake of fatty acids and mitochondrial *β*-oxidation (34). At the same time, the stabilization of hypoxia-inducible factor 1-alpha (HIF-1*α*) will upregulate glucose transporters as well as key glycolytic enzymes, including the expression of hexokinase (35). Therefore, myocardial cells increasingly depend on glycolysis. This phenomenon is often described as a return to a “fetal-like” metabolic state (34, 36). Preclinical animal models have already supported this metabolic switch. In this dog model, changes in biochemical markers are directly related to imaging results, and changes in ¹⁸F-FDG uptake can be seen even before any visible structural or functional remodeling of the heart occurs (37).

This adaptation may initially help maintain energy supply under some conditions. However, it is less efficient and may become maladaptive over time. More importantly, this metabolic shift directly affects PET imaging, but the pattern of ^18^F-FDG uptake may vary depending on the different stages of RIHD. In the early inflammatory stage, radiation-induced endothelial damage, hypoxia-related signals, and inflammatory cell recruitment may increase local glucose utilization, resulting in elevated or heterogeneous myocardial ^18^F-FDG uptake (18, 38, 39). However, as the injury progresses to the fibrotic stage, with severe myocardial cell damage, sparse microvasculature, and myocardial cell fibrosis, FDG uptake in severely affected areas may decrease due to the reduced number of surviving myocardial cells.

### 3.4 Inflammation and immune activation

Radiation-induced damage is also accompanied by persistent inflammatory reactions (40). Injured cells release damage-associated molecular patterns (DAMPs), which will activate the innate immune pathways and promote the recruitment of inflammatory cells (41, 42). Activated immune cells, especially macrophages, undergo metabolic reprogramming and tend to support rapid cytokine production via glycolysis (43). This forms a feedback cycle in which inflammation will further aggravate metabolic disorders and tissue damage.

From the perspective of molecular imaging, inflammatory activity is another important factor that changes myocardial metabolism. Increased uptake of metabolic tracers in affected regions may reflect cardiomyocyte changes and inflammatory cell infiltration (18, 44, 45).

### 3.5 Fibrosis and late structural remodeling

In the later stages of RIHD, persistent damage and inflammation promote fibroblast activation and extracellular matrix deposition, resulting in myocardial fibrosis (9, 16, 46). Transforming growth factor-*β* (TGF-*β*) plays a pivotal role in this process by promoting differentiation into myofibroblasts (47). Fibrotic remodeling will lead to myocardial hardening and impaired cardiac function (48, 49). Compared with earlier stages, metabolic activity in fibrotic tissue may be reduced; however, the transition from active injury to fibrosis can be heterogeneous (16, 50). This dynamic process underscores the need for imaging methods that can capture different stages of disease progression. In this case, PET tracers for metabolism, perfusion, or fibrosis may provide complementary information.

## 4 PET imaging in RIHD

Based on the mechanistic alterations described above, particularly the shift in myocardial metabolism and the progression from inflammation to fibrosis, PET has emerged as a promising tool for detecting RIHD at different stages. Unlike conventional imaging modalities that primarily detect structural changes, PET provides functional and molecular-level information, making it particularly suitable for identifying early and potentially reversible abnormalities (51). Given the complex and dynamic nature of RIHD, no single imaging modality can fully detect all aspects of the disease (52). The key PET tracers used in RIHD assessment are summarized in Table 2.

**Table 2:** Summary of PET tracers in RIHD assessment.

Tracer	Molecular Pathway	Pathophysiological Relevance in RIHD	Imaging Finding in RIHD	Representative References
¹⁸F-FDG	Glucose metabolism	Detects shift from fatty acid oxidation to glycolysis (metabolic reprogramming); marks inflammatory cell infiltration	Increased or heterogeneous myocardial FDG uptake may be observed during early stage after thoracic radiotherapy, whereas reduced uptake may occur in late stage with cardiomyocyte loss or fibrotic replacement	Dreyfuss et al. (48) Sha et al. (98) Wei et al. (68)
¹³N-ammonia	Myocardial perfusion	Assesses microvascular dysfunction due to endothelial injury and capillary rarefaction	Reduced myocardial blood flow and coronary flow reserve in irradiated myocardium	Wu et al. (63)
18F-FAPI	Fibroblast activation protein	Shows activated fibroblasts, an early step in fibrotic remodeling before irreversible fibrosis	Increased cardiac FAPI uptake in patients post-radiotherapy, potentially identifying an active pro-fibrotic state	Wei et al. (68)

### 4.1 Assessment of myocardial glucose metabolism: ^18^F-FDG PET

^18^F-FDG PET is currently the most commonly used radiotracer in evaluating myocardial metabolic activity (53). Several studies have reported that even in the absence of obvious dysfunction, the irradiated myocardium will have a heterogeneous or diffuse increase in FDG uptake (54–56). This shows that metabolic changes may occur before structural remodeling and may become a biomarker of early imaging. However, the level of FDG uptake cannot be used as the sole criterion for assessing the severity of RIHD. An increase in FDG uptake is more likely to reflect early inflammatory activity, hypoxia-driven glycolysis, or metabolic remodeling, while a decrease in FDG uptake may occur in areas where metabolically active heart cells are lost or replaced by fibrosis. Therefore, it's better to interpret FDG uptake based on how it changes over time.

In addition, interpreting FDG uptake in the heart is not easy. Physiological myocardial glucose utilization varies with diet and metabolic conditions, which may lead to differences in imaging results (57, 58). Therefore, although ^18^F-FDG PET is highly sensitive to early changes, its specificity remains limited. It is important to carefully prepare patients and standardized imaging protocols (59).

### 4.2 Myocardial perfusion imaging

In addition to metabolic imaging, PET-based myocardial perfusion imaging can also provide important information about the microvascular function (60). Tracers such as ^13^N-ammonia and Rubidium-82 (^82^Rb) can be used to quantitatively evaluate myocardial blood flow and coronary flow reserve (61).Given that endothelial injury and microvascular rarefaction are early events in RIHD, perfusion abnormalities may develop before significant coronary artery stenosis becomes evident (21, 62, 63). Studies have demonstrated that reduced myocardial perfusion reserve can be detected in patients after thoracic radiotherapy, reflecting impaired microvascular function (63–65).

Compared with FDG imaging, perfusion PET offers better specificity for vascular dysfunction; however, it may be less sensitive to early metabolic changes (60, 65). In this case, combining perfusion imaging with metabolic evaluation may be able to more comprehensively assess cardiac damage caused by radiation.

### 4.3 Imaging of fibroblast activation and fibrosis: FAPI PET

Recently, PET imaging based on FAPI has attracted attention as a new method of visualizing cardiac remodeling (66, 67). FAPI tracer targets activated fibroblasts, which play a central role in myocardial fibrosis. A study has shown that increased FAPI uptake correlates with fibroblast activation and extracellular matrix remodeling, even at stages when fibrosis is not yet apparent on conventional imaging (68). In the background of RIHD, FAPI PET may allow us to discover the ongoing fibrosis processes at an earlier stage. Compared with FDG, the myocardial background uptake of FAPI imaging seems to be lower, which may improve the detectability of lesions and facilitate interpretation (69). In addition, since normal myocardial cells do not have physiological FAPI accumulation, there is no need for complicated dietary preparation and prolonged fasting, which greatly simplifies the clinical procedure (70, 71). Despite the prospects, there are still limitations before wider clinical application. One is that FAPI uptake is not exclusively specific to radiation injury, it may also be elevated in age-related interstitial fibrosis, ischemic scars, or uptake by concurrent tumors, making the imaging more difficult to interpret. In addition, the optimal timing for imaging after radiotherapy has not yet been determined (72). Its clinical application is still under development, and further research is needed to establish standardized protocols and verify its prognostic value (73).

## 5 Clinical evidence of PET imaging in RIHD

Based on the mechanistic basis and imaging characteristics discussed above, the current clinical studies provide more and more evidence that PET imaging can detect RIHD at different stages (18, 54–56, 74, 75). Although the number of available studies is still limited, there has been a consistent pattern on metabolic changes, microvascular dysfunction and early fibrosis reshaping in patients after radiation therapy.

### 5.1 ^18^F-FDG PET in clinical studies

Among the available tracers, ^18^F-FDG PET has been the most widely investigated in patients with suspected RIHD (53). Several clinical studies have reported increased or heterogeneous myocardial FDG uptake following thoracic radiotherapy, even in asymptomatic patients (54–56, 74, 75). Table 3 summarizes the main characteristics, timing of PET use, key findings, and limitations of clinical studies published between 2021 and 2026.

**Table 3:** Summary of Key clinical PET studies in RIHD.

Author	Cancer Type (*N*)	Tracer Used	Timing of PET	Key Findings	Limitations
Chau et al. (54)	Left-sided Breast Cancer (*N* = 15)	18F-FDG	Baseline and 1 month post-RT	Significant increase in 18F-FDG SUVmean in LAD territory (10%, *P* = 0.04). Significant increase in ECV at apex (6%) and base (5%). Significant reduction in stroke volume (−7%).	Small sample size; Short-term follow-up; Results not correlated with hard clinical cardiac events.
Won et al. (56)	Left-sided Breast Cancer (*N* = 16)	18F-FDG	Baseline, one week after RT(Post-1), and at 12, 24, and 48 weeks (Post-2, Post-3, and Post-4).	Significant increase in FDG uptake ratio (irradiated vs. non-irradiated) in regions ≥30 Gy, persisting up to 1 year. Markedly decreased GLS_LAX at 12 weeks.	Small sample size; No cardiac MRI validation; Routine fasting only (suboptimal myocardial suppression).
Bütof et al. (75)	Esophageal Cancer (*N* = 125)	18F-FDG	Baseline and during the last week of neoadjuvant radiochemotherapy	The heart mean dose is significantly associated with worse OS (*P* = 0.005). PET-derived cardiac metabolic parameters were not predictive of survival in this cohort.	Retrospective design; Heterogeneous RT techniques and nodal irradiation protocols; Standard oncologic FDG protocol without dedicated myocardial suppression.
Zakem et al. (74)	Esophageal Cancer (*N* = 51)	18F-FDG	Baseline and post-RT (median 56 days)	Change in cardiac SUVmean was a predictor of OS (*P* = 0.028). A dose-response trend was observed with an average increase of 0.044 SUV per 10 Gy, suggesting potential prognostic value of longitudinal cardiac metabolic changes.	Retrospective; Small cohort; Variable post-RT scan timing; Standard oncologic FDG protocol without dedicated myocardial suppression.
Cho et al. (55)	Stage III NSCLC (*N* = 133)	18F-FDG	Early post-concurrent chemoradiation therapy (median 11 days)	Post-RT LV SUVmax (>12.84) independently predicted cardiac events (≥grade 2), particularly in patients with higher mean heart dose (>11.1 Gy).	Retrospective; Majority of events were grade 2 (pericardial effusion); Baseline cardiac status not fully characterized.

Chau et al. (54) found a significant 10% elevation in mean standardized uptake value (SUVmean) in the left anterior descending (LAD) coronary artery territory as early as 1 month after radiotherapy for left-sided breast cancer (*P* = 0.04), accompanied by increased extracellular volume and reduced stroke volume, while LVEF remained preserved. This finding shows that ^18^F-FDG PET can detect acute inflammation and early functional changes when cardiac function is still normal and there are no structural changes. Similarly, Won et al. (56) found that the FDG uptake ratio in myocardium exposed to >30 Gy radiation was significantly elevated at 3 months post-radiotherapy and persisted up to 1 year. At the same time, patients remained asymptomatic throughout the follow-up period. This suggests that metabolic abnormalities can persist in the subclinical stage over the long term. These clinical findings mainly testify to the metabolic changes in the early or subacute stages after radiotherapy. However, they should not be considered representative of all stages of RIHD. The level or direction of FDG uptake may depend on the balance between active inflammation, adaptive glycolytic metabolism, the amount of surviving myocardial cells, and fibrotic replacement.

Although Bütof et al. (75) did not find that PET parameters could predict overall survival (OS) in patients with esophageal cancer, they confirmed that mean heart dose (MHD) was significantly associated with survival, highlighting the critical importance of radiation dose control. In contrast, Zakem et al. (74) demonstrated for the first time that the change in myocardial SUVmean between pre- and post-radiotherapy was an independent predictor of OS (*P* = 0.028), with a clear dose-dependent trend. The differences between these results may be related to different research designs rather than contradictory paradoxes. In the study by Bütof et al. (75), ^18^F-FDG PET was performed before treatment and during the last week of neoadjuvant chemoradiotherapy, and the study subjects were a relatively large group of patients planned for triple therapy. In this case, dose parameters of the heart and lungs may have a greater impact on survival than the metabolic parameters derived from PET. By comparison, Zakem et al. (74) focused on the longitudinal changes in cardiac FDG uptake between pre- and post-treatment PET scans in a smaller cohort, which had greater variability in disease stage, surgical status, radiation dose, and intervals between post-treatment imaging. In addition, the protocols of these two studies primarily used tumor FDG PET protocols rather than protocols specifically designed for cardiac inflammation, so differences in myocardial physiological uptake suppression and background signals may have affected the measurement of cardiac standardized uptake value (SUV). The statistical models used in these two studies also differ. Bütof et al. (75) evaluated PET parameters together with blood values and normal tissue dose metrics, whereas Zakem et al. (74) specifically modeled changes in cardiac SUV as a longitudinal biomarker, while adjusting for selected clinical and treatment-related factors. Therefore, the prognostic value of cardiac metabolic changes derived from FDG PET, as shown by Bütof et al. (75), cannot be completely dismissed. In contrast, these experimental results suggest that the prognostic performance of FDG PET may depend on the timing of imaging, myocardial metabolic preparation, patient population, treatment background, and statistical modeling. At present, cardiac metabolic biomarkers derived from FDG PET should be considered promising but still exploratory, and their prognostic value needs to be validated in prospective studies using standardized cardiac imaging protocols. Furthermore, Cho et al. (55) found that a left ventricular (LV) maximum standardized uptake value (SUVmax) exceeding 12.84 was an independent predictor of grade ≥2 cardiac events (hazard ratio=2.14, *P* = 0.018) as early as 11 days after chemoradiotherapy for NSCLC, with a particularly pronounced effect in patients receiving high cardiac radiation doses. In summary, these studies indicate that cardiac metabolic changes obtained based on FDG PET may have potential value in the early detection of myocardial injury and risk stratification. However, the prognostic significance of these findings remains uncertain and needs to be validated in prospective studies using standardized cardiac FDG-PET protocols.

¹⁸F-FDG PET has demonstrated robust potential to detect metabolic reprogramming in RIHD across multiple cancer types and different periods. However, its clinical translation is limited by different methods, and there is an urgent need to formulate a standardized image protocol and carry out prospective multi-center validation. This standardized protocol can help eliminate the methodological differences that trouble the field, allow the results of different research institutions to be directly compared, and lay a solid foundation for the establishment of evidence-based diagnostic thresholds and clinical practice guidelines. But even if there is a standardized protocol, ¹⁸F-FDG PET still has its limitations and can't be used only to evaluate imaging examination of heart problems.

### 5.2 PET assessment of myocardial perfusion

Compared with metabolic imaging, PET-based myocardial perfusion imaging still has relatively limited clinical research evidence in RIHD. Myocardial perfusion PET might offer a unique perspective for assessing radiation-related coronary microvascular dysfunction, which is increasingly recognized as an important early component of RIHD (76). Wu et al. (63) using quantitative ¹³N-ammonia PET have shown that even if the patients do not have severe obstructive coronary artery disease, there is significant impairment of corrected coronary flow reserve (cCFR) in people with left ventricular dysfunction. The incidence of decreased cCFR in these patients is much higher, and the reduction of cCFR is an independent influencing factor of major adverse cardiovascular events (MACE) during follow-up. It is worth noting that those patients whose LVEF and cCFR are at normal levels did not have MACE, which highlights the potential prognostic value of evaluating coronary microvascular function. The above study confirms that during the development of RIHD, coronary microvascular dysfunction occurs relatively early, earlier than obvious epicardial coronary artery lesions and severe cardiac structural abnormalities. This is different from conventional perfusion defects caused by large vessel stenosis. In most case, a decrease in coronary flow reserve (CFR) means diffuse endothelial dysfunction and abnormal microvascular vasodilatation (77). Therefore, quantitative perfusion of PET can detect functions related to myocardial blood flow regulation and obtain relevant information, while traditional anatomical imaging methods cannot fully obtain this information when used alone.

It is worth noting that although myocardial perfusion imaging and metabolic imaging reflect the different pathophysiological mechanisms of RIHD, they can complement each other (76). The increase in FDG uptake mainly indicates disordered glucose metabolism and active inflammatory reactions, while myocardial blood flow deficiency and decreased CFR can more directly reflect abnormal vascular function and microcirculation damage (76, 77). This difference has great clinical significance, because endothelial damage and microvascular dysfunction caused by radiation are the core mechanisms that subsequently lead to myocardial fibrosis, ischemia and ventricular remodeling.

Although perfusion PET is less sensitive than metabolic imaging in identifying the earliest subclinical cardiac lesions, quantitative measurements of myocardial blood flow and CFR may can more accurately assess vascular damage caused by radiation (78). In addition, the combined integration of perfusion PET and metabolic PET-related indicators may simultaneously evaluate inflammatory metabolic remodeling and microvascular functional damage, thus providing a more comprehensive analysis of the condition of RIHD (77).

### 5.3 Emerging evidence of FAPI-PET

FAPI PET, as an emerging imaging technology, may provide important evidence for implementing individualized cardiovascular risk stratification in patients after receiving RT (79). Traditional cardiac surveillance strategies mainly rely on LVEF and indicators of structural abnormalities to make assessments. However, when these indicators appear, they usually indicate that myocardial damage is irreversible and the opportunity for timely intervention is missed (80). In contrast, relying on the metabolic levels and FAPI PET can present early metabolic changes in the myocardium and fibroblast-related pathological changes, thereby enabling earlier detection of subclinical myocardial remodeling (68). This can help clinicians implement interventions earlier and make personalized follow-up plans. Wei et al. (68) had shown that in patients with esophageal squamous cell carcinoma undergoing RT, myocardial FAPI uptake significantly increased. The tissue-to-blood ratio rose from 1.53 ± 0.53 before treatment to 1.88 ± 0.70 during chemoradiotherapy, with the difference being statistically significant (*P* = 0.015). In a constructed rat model of RIHD, myocardial FAPI uptake was significantly elevated by the second week after irradiation, reaching a peak at the fifth week at 2.63 ± 0.07%ID/mL, compared to 0.87 ± 0.07%ID/mL in the control group (*P* < 0.05). However, LVEF in the rats did not show significant changes until the eighth week of post-irradiation. These results indicate that fibroblast activation and myocardial metabolic remodeling occur much earlier than clinically detectable cardiac functional damage, and the animal model also confirms that FAPI PET imaging may be expected to identify high-risk patient populations before irreversible cardiac dysfunction develops. The key is that the spatial distribution of fibroblast activation protein uptake in myocardial tissue closely corresponds to the areas receiving high-dose radiation, indicating that FAPI PET imaging can reflect local myocardial tissue remodeling and microvascular damage caused by RT. Moreover, by the fifth week post-irradiation, FDG uptake in the damaged myocardial regions decreased to 2.99 ± 0.08%ID/mL, compared with 6.04 ± 0.07%ID/mL in normal regions (*P* < 0.05). This kind of reduced FDG uptake should be interpreted in conjunction with the stage of the disease and the survival of tissue cells. Unlike early clinical studies where increased FDG uptake may reflect acute inflammation and metabolic changes, the reduced FDG uptake in the myocardial regions damaged at week 5 in the animal model of this experiment may indicate more severe local injury, fewer surviving cardiomyocytes, lower density, impaired substrate utilization, and progressive fibrosis. In this case, increased FAPI uptake and decreased FDG uptake may be complementary rather than contradictory. FAPI can identify activated fibroblasts and active fibrotic remodeling, while reduced FDG uptake may reflect the loss of metabolically active myocardium in severely damaged regions. Therefore, the combination of FDG and FAPI may help distinguish early inflammation and metabolic remodeling from late tissue damage caused by fibroblasts.

These quantitative research results show that from the various indicators obtained by PET scans may have the potential to become imaging biomarkers for assessing RIHD and classifying the prognosis of disease. Another clinical value of PET imaging is that it can carry out long-term dynamic monitoring of RIHD, since RIHD is a dynamic and continuously developing pathological process, symptoms may continue to develop for several years or even decades after treatment (79).

## 6 Challenges and future directions

To transform the theoretical potential of PET imaging in RIHD into daily clinical applications, several major obstacles need to be overcome. With the development of cardiac oncology towards precise monitoring, these current limitations must be addressed through a unified approach, including technical standardization, reliable clinical trials and multi-mode collaboration. An international consensus proposal has explicitly stated that the validation standards for PET scanners, including phantoms, methods, and acceptance criteria, are currently not uniform across clinical trials and institutions, and further proposes a standardized PET scanner validation paradigm to ensure quantitative accuracy and reproducibility (81).

### 6.1 Overcoming physiological interference and standardizing protocols

Normal myocardium has significant metabolic flexibility and can switch between the use of fatty acids and glucose depending on different diets, fasting states, drug use, insulin function levels and systemic metabolic conditions. This physiological regulation is a major obstacle to the application of cardiac ^18^F-FDG PET in RIHD, because non-specific myocardial glucose uptake can obscure subtle radiation-related inflammation or metabolic abnormalities, and it may also reduce the comparability of scans across different institutions. Therefore, standardized patient preparation should be regarded as a fundamental prerequisite for future RIHD research using ^18^F-FDG PET. For cardiac ^18^F-FDG PET protocols specifically used to detect radiation-related myocarditis or focal metabolic injury, a myocardial suppression strategy that can be verified and reproduced should be specified and reported. A concise and reproducible checklist for protocol and reporting requirements is provided in Table 4. Based on the existing PET protocols for heart inflammation and cardiac sarcoidosis, a feasible and practical framework might include: (1) Follow a high-fat, low-carbohydrate, or no-carbohydrate diet within 24-48 h before imaging examinations, or eat at least two high-fat, very low-carbohydrate meals the day before the examination, with each meal containing more than 35 g of fat and less than 3 g of carbohydrates; (2) Fast for at least 12 h before injecting the tracer, preferably 12-18 h. If adherence to dietary restrictions is uncertain, fasting for more than 18 h is also an option; (3) Avoid strenuous exercise for at least 12 h before the scan; (4) Before injecting the tracer, one should avoid consuming foods, drinks, and medications containing carbohydrates, and avoid intravenous injection of drugs; and (5) measurement of blood glucose before ^18^F-FDG injection, ideally with glucose <11 mmol/L or <180 mg/dL (82–84). The imaging report should document the patient's compliance, precise fasting duration, blood glucose levels, diabetes status, use of insulin or steroids, and any use of medications containing carbohydrates to facilitate assessment and reproducibility (82, 83). Intravenous unfractionated heparin may be used as an auxiliary method for dietary preparation, but it should not replace dietary restrictions and prolonged fasting regimens, as its additive effect on myocardial suppression remains unclear. If used and not contraindicated, the commonly reported protocol is a single intravenous bolus of unfractionated heparin at 50 IU/kg approximately 15 min before ^18^F-FDG administration (82–84). Patients with bleeding risk, thrombocytopenia, recent surgical history, or other contraindications should avoid using heparin or use it with caution. In addition, the condition of patients with poor myocardial suppression effect should be truthfully recorded; for imaging results showing diffuse and uniform uptake in the left ventricle, careful interpretation is required, and re-examination can be arranged if necessary. Even when following the protocol, a small number of patients may still experience poor myocardial suppression, which highlights the importance of implementing quality control and standardized, transparent reporting (84).

**Table 4:** Proposed standardized ^18^F-FDG PET protocol and reporting framework for RIHD.

Protocol component	Recommended parameters for myocardial FDG suppression or acquisition	Reporting and interpretation requirements
Dietary preparation	Use a high-fat, low-carbohydrate or no-carbohydrate diet for 24–48 h before imaging, or at least two high-fat, very-low-carbohydrate meals on the day before PET; each meal may contain >35 g fat and <3 g carbohydrates (82–84).	Record the preparation strategy, exact duration, dietary compliance, and any protocol deviations. The purpose is to shift myocardial substrate use toward fatty acids and reduce nonspecific physiological FDG uptake.
Fasting before tracer injection	Fast for at least 12 h before 18F-FDG injection; a 12–18 h fast is preferred. If dietary adherence is uncertain, fasting for >18 h can be considered (82–84).	Report the precise fasting duration. Inadequate fasting should be flagged because it may reduce the specificity of myocardial FDG interpretation.
Exercise, carbohydrate exposure, and intravenous glucose	Report the precise fasting duration. Inadequate fasting should be flagged because it may reduce the specificity of myocardial FDG interpretation (82–84).	Document recent exercise, carbohydrate exposure, medication use, and intravenous fluid administration, because these factors may increase nonspecific myocardial FDG uptake.
Blood glucose and metabolic status	Measure blood glucose before 18F-FDG administration; an ideal target is <11 mmol/L or <180 mg/dL. Document diabetes status and recent insulin or steroid use (82, 83).	Report glucose level, diabetes status, insulin or steroid use, and related metabolic conditions to improve reproducibility and cross-center comparability.
Optional heparin adjunct	Intravenous unfractionated heparin may be used as an adjunct, not as a substitute for diet and fasting. If used and not contraindicated, a commonly reported protocol is 50 IU/kg as a single intravenous bolus approximately 15 min before 18F-FDG injection (82–84).	Report heparin dose, timing, and contraindication screening. Avoid or use caution in patients with bleeding risk, thrombocytopenia, recent surgery, or other contraindications. The additive effect remains uncertain.
Quality control for myocardial suppression	Assess the pattern of background myocardial uptake before interpreting RIHD-related abnormalities. Diffuse and homogeneous left ventricular uptake indicates poor suppression and should be interpreted cautiously; repeat imaging may be considered if necessary (84).	Report suppression quality, whether uptake is diffuse, focal, regional, or heterogeneous, and whether results are suitable for evaluating radiation-related focal metabolic injury.
Quantitative acquisition and motion analysis	Static SUV-based analysis can be used, but dynamic 18F-FDG PET with Patlak graphical analysis should be considered in longitudinal or multicenter studies when feasible. Patlak-derived Ki provides a more quantitative index of FDG metabolic trapping than SUV alone and can be paired with glucose correction (85).	Report acquisition mode, scan timing, arterial or image-derived input function, Ki, glucose-corrected metabolic rate when available, and the rationale if only static SUV is used.
Minimum image-analysis and reporting dataset	At minimum, report injected dose, uptake time, dietary preparation, fasting duration, blood glucose, reconstruction parameters, attenuation correction method, region-of-interest definition, and the metrics used.	Specify whether SUVmean, SUVmax, target-to-background ratio, Patlak-derived Ki, glucose-corrected metabolic rate, or 17-segment left ventricular analysis was applied. These details support multicenter reproducibility and linkage with radiation dose, perfusion, FAPI uptake, and cardiovascular outcomes.

In addition to patient preparation, quantitative acquisition and analysis of information should also be standardized. Caution should be exercised when using SUV-based parameters, as SUV may be affected by uptake time, differences in body structure, blood glucose levels, scanner calibration, reconstruction parameter settings, attenuation correction, and residual blood-pool activity. Dynamic ^18^F-FDG PET with Patlak graphical analysis can provide the influx rate constant Ki, which represents a more quantitative index of ^18^F-FDG metabolic trapping than SUV alone (85). Patlak analysis requires dynamic imaging and an arterial or image-derived input function, but it can help separate irreversibly trapped, metabolized FDG from reversible blood-pool and unmetabolized FDG components (85). Therefore, future research on RIHD could consider using the Ki derived from Patlak for dynamic acquisition, and, where feasible, especially in longitudinal or multicenter studies, using glucose-corrected glucose metabolism rates. But at a minimum, the study should report the injected dose, uptake time, dietary preparation method, fasting duration, blood glucose levels, reconstruction parameters, attenuation correction method, delineation of regions of interest, and whether SUVmean, SUVmax, target-to-background ratio, Patlak-derived Ki, or 17-segment left ventricular analysis parameters were used. This standardized report can improve the reproducibility of multicenter studies and enable FDG-PET biomarkers to be more reliably associated with radiation dose distribution, impaired perfusion, fibroblast activation, and subsequent clinical cardiovascular outcomes.

### 6.2 Bridging the evidentiary Gap with prospective longitudinal cohorts

Although PET is highly sensitive to early metabolic changes, integrating it into routine clinical monitoring remains limited because most existing evidence comes from retrospective, single-center studies and surrogate imaging endpoints. Therefore, the existing guidelines still give priority to echocardiography and CMR because of their high availability and extensive long-term verification (86). If we want to turn PET from an experimental tool into a part of post-treatment care recommendations, we really need large, prospective, multicenter longitudinal cohort studies. These studies must clearly link the metabolic changes derived from early PET with actual clinical results, such as the incidence of Major Adverse Cardiovascular Event. However, it should be emphasized that initiating specific cardioprotective treatments, such as angiotensin-converting enzyme inhibitors (ACEIs) or sodium-glucose cotransporter 2 (SGLT2) inhibitors, based solely on information obtained from PET is still at the research stage. Although theoretically promising, the existing clinical evidence for PET-guided therapeutic interventions is very limited. Therefore, it is highly necessary to conduct prospective randomized trials in the future to verify whether treatment initiated based on early metabolic signals can effectively alter disease progression and reduce long-term cardiovascular mortality.

### 6.3 Advancing risk stratification through hybrid imaging and artificial intelligence

With the development of cardiac oncology, overcoming diagnostic limitations will increasingly depend on technological integration and advanced computational analysis. The growing popularity of the integrated PET/MRI platforms represents a great shift, which allows us to obtain metabolic, functional, and structural data at the same time in a single scan (87). Although PET can detect early molecular abnormalities, like metabolic changes or fibroblast activation, the simultaneous CMR sequences can provide high-resolution tissue features, helping doctors clearly distinguish potentially reversible acute inflammatory edema from irreversible chronic interstitial fibrosis. In addition, beyond traditional visual interpretation, radiomics and AI have huge potential in pulling out complex quantitative features from these images. However, it is necessary to strictly distinguish between mature clinical application methods and cutting-edge research directions that are still in the exploratory stage. Although PET/MRI has gradually become widespread, the application of radiomics and artificial intelligence in RIHD is still in its infancy. At present, there is very little evidence to support the clinical use of predictive models constructed with AI in this field. Incorporating texture features along with clinical indicators and dose distribution maps into machine learning models is a highly promising research direction. Such technologies still require a large number of prospective clinical trials for validation before they can meet clinical standards and promote a shift in cardiovascular diagnosis and treatment from passive symptomatic treatment to proactive prevention.

### 6.4 Expanding PET tracers for mechanistic validation

Although current PET evidence related to RIHD mainly involves ^18^F-FDG, perfusion imaging, and FAPI, data on RIHD regarding fatty acid metabolism, hypoxia, and inflammation tracers targeting macrophages are still very limited. This gap is significant because FDG uptake alone cannot fully verify the proposal of a shift from fatty acid oxidation to glucose utilization. Future research on RIHD could consider using fatty acid tracers such as ^11^C-palmitate or ^18^F-fluoro-6-thia-heptadecanoic acid (^18^F-FTHA). These tracers have been used in cardiac-related studies of non-radiation-induced heart disease to assess myocardial long-chain fatty acid metabolism and myocardial fatty acid uptake levels (88, 89). Hypoxia-targeted tracers also have application value because endothelial damage and microvascular rarefaction caused by radiotherapy can activate hypoxia-related pathways and promote glycolytic metabolism (90). ^18^F-Fluoromisonidazole (^18^F-FMISO) has been used to detect hypoxic but still viable myocardium in ischemic heart disease, suggesting that it may help determine whether local FDG abnormalities in future RIHD studies are related to hypoxia-driven metabolic adaptation (91, 92). In addition, non-FDG inflammatory tracers may help differentiate between inflammatory cell activity and myocardial cell glucose utilization. ^68^Ga-DOTATATE targets somatostatin receptor subtype 2 expressed by activated macrophages and has been used to image post-infarction myocardial inflammation (93). Therefore, in future RIHD research, it may provide a signal that is more specific to macrophages than FDG.

Currently, these tracers should be regarded as candidates rather than established imaging tools for RIHD. Incorporating them into future longitudinal PET studies may help determine whether radiation-related FDG changes reflect substrate conversion, hypoxia, macrophage infiltration, or downstream fibroblast activation.

### 6.5 Radiation exposure and safety considerations in PET imaging

Although PET imaging provides valuable metabolic information for the early detection of RIHD, the radiation burden associated with repeated PET/CT examinations must be carefully considered. PET/CT examination involves two sources of radiation: radiation from the injected radioactive tracer and radiation exposure from the CT scan (94). Therefore, repeated scanning at baseline, early after radiotherapy, and during long-term follow-up may lead to cumulative radiation exposure, especially when PET/CT is included in longitudinal monitoring protocols (95). This issue is particularly relevant for long-term cancer survivors with good prognostic outcomes, such as patients with breast cancer or lymphoma, who may live for many years after radiotherapy and may have already received substantial therapeutic radiation exposure (96). From the perspective of radiation protection, continuous PET/CT examinations should follow the principles of justification and optimization. PET imaging should not be used as a routine follow-up for all patients after chest RT. In contrast, it should be used based on risk adjustment and clinical rationalization according to baseline cardiovascular risk, mean heart dose, cardiac substructure dose, symptoms, biomarkers, abnormalities in echocardiography or cardiac MRI, and the expected impact of PET results on clinical management. For patients with low baseline risk and no abnormalities detected during routine cardiac monitoring, echocardiography, cardiac magnetic resonance, and serum biomarkers may still be more suitable for routine follow-up, while PET should be used for high-risk patients, cases with unclear conditions, or prospective scientific research (80).

There are several strategies to reduce the radiation burden from continuous PET monitoring. First, when PET/CT is used solely for the molecular assessment of myocardial injury, it is best to use only low-dose CT for attenuation correction and anatomical localization, rather than diagnostic CT, unless the clinician requires diagnostic CT information. Secondly, the PET acquisition protocol should use the minimum activity of the radiotracer that can meet image quality and provide quantitative reliability, especially when using modern digital PET systems, time-of-flight reconstruction, point spread function modeling, or long axial field-of-view scanners. Third, the administered activity, scan range, CT dose, dose-length product, and the estimated effective doses for PET and CT should be recorded in the imaging diagnostic report and research protocol to track cumulative radiation exposure in longitudinal studies. For certain patients, PET/MRI may be a crucial alternative because it can provide information on the heart's metabolism, function, and structure while eliminating the radiation dose produced by CT (97). This advantage is particularly beneficial for young patients who may need repeated exposure to radiation and cancer survivors with a good long-term prognosis. However, PET/MRI is not commonly available in all hospitals or laboratories because it is expensive and there is still radiation exposure from the PET radioactive tracers themselves (95). Therefore, whenever feasible, PET/MRI should be considered as a strategy to reduce radiation dose, but it should not be regarded as a complete solution to the problem of radiation exposure related to radioactive tracers.

Finally, the timing of positron emission tomography examinations needs to be carefully planned. Future research on RIHD should not uniformly adopt frequent examination intervals for all patients, but should develop differentiated examination intervals based on risk stratification. For example, baseline PET scans can be used in high-risk groups or research protocols, while early follow-up PET scans may only be used for patients with higher radiation doses to the heart, abnormal biomarkers, or functional changes. When the initial PET results are negative or clinically stable, longer scan intervals or non-ionizing follow-up examination protocols should be prioritized. This approach can minimize unnecessary radiation exposure while maintaining the prognostic value of PET and improving the ethical acceptability of PET-based monitoring for RIHD (80).

## 7 Conclusion

RIHD remains a great threat to the long-term survival of cancer patients. This review highlights that metabolic reprogramming is a sentinel event in the pathophysiology of RIHD, preceding macro-structural damage and functional decline. Molecular imaging via PET provides a unique and powerful tool for this early biochemical change. Despite the challenges in physiological background and standardized needs, new tracers, mixed the integration of PET/MRI system and artificial intelligence provides a promising path. As we transition into an era of precision cardio-oncology, PET imaging is expected to evolve from a professional research tool into a cornerstone of clinical monitoring, enabling to achieve early detection, risk stratification, and the timely intervention, so as to improve the cardiovascular health of cancer survivors around the world.
