# Supplementary material for: Metabolic reprogramming in radiation-induced heart disease: the emerging role of PET imaging
Source: Front Cardiovasc Med. 2026 Jul 23;13:1883964. doi: 10.3389/fcvm.2026.1883964 (PMC13441930; doi:10.3389/fcvm.2026.1883964)
Supplement: Supplementary file 1 [file Datasheet1.docx]

**Supplementary Material 1**

Detailed Database-Specific Search Strategies:

**Pubmed:**

(radiotherapy[Title/Abstract] OR radiation[Title/Abstract] OR irradiation[Title/Abstract]) AND (heart[Title/Abstract] OR cardiac[Title/Abstract] OR myocardial[Title/Abstract] OR myocardium[Title/Abstract] OR cardiotoxicity[Title/Abstract]) AND (PET[Title/Abstract] OR "positron emission tomography"[Title/Abstract] OR "molecular imaging"[Title/Abstract] OR tracer[Title/Abstract] OR radiotracer[Title/Abstract] OR probe[Title/Abstract] OR FDG[Title/Abstract] OR FAPI[Title/Abstract] OR palmitate[Title/Abstract])

Publication date: January 2021- April 2026

Results: 305 publications

**Web of science:**

TS=(radiotherapy OR radiation OR irradiation)

AND TS=(heart OR cardiac OR myocardial OR myocardium OR cardiotoxicity)

AND TS=(PET OR "positron emission tomography" OR "molecular imaging" OR tracer OR radiotracer OR probe OR FDG OR FAPI OR palmitate)

AND PY=(2021-2026)

Results: 64 publications

**Cochrane:**

(radiotherapy OR radiation OR irradiation) AND (heart OR cardiac OR myocardial OR myocardium OR cardiotoxicity) AND (PET OR "positron emission tomography" OR "molecular imaging" OR tracer OR radiotracer OR probe OR FDG OR FAPI OR palmitate)

Publication date: January 2021- April 2026

Results: 35 publications

**Euorpe PMC :**

(TITLE_ABS:"radiotherapy" OR TITLE_ABS:"radiation" OR TITLE_ABS:"irradiation") AND (TITLE_ABS:"heart" OR TITLE_ABS:"cardiac" OR TITLE_ABS:"myocardial" OR TITLE_ABS:"myocardium" OR TITLE_ABS:"cardiotoxicity") AND (TITLE_ABS:"PET" OR TITLE_ABS:"positron emission tomography" OR TITLE_ABS:"molecular imaging" OR TITLE_ABS:"tracer" OR TITLE_ABS:"radiotracer" OR TITLE_ABS:"probe" OR TITLE_ABS:"FDG" OR TITLE_ABS:"FAPI" OR TITLE_ABS:"palmitate")

Publication date: January 2021- April 2026

Results: 300 publications

Total publications:704 publications

**Inclusion Criteria:**

1. Patients with malignancies who received radiotherapy targeting the thoracic or mediastinal region.
2. Definitive radiotherapy intervention, administered either alone or in combination with chemotherapy.
3. Evaluation of alterations in myocardial metabolic uptake must be performed using PET or PET/CT imaging.

**Timeframe:** Literature published within the past five years (2021–2026).

**Exclusion Criteria:**

1. Case reports
2. conference abstracts
3. reviews
4. studies that do not use core cardiovascular imaging indicators as study endpoints
